# Supplementary material for: Analysis of Usage Data from a Self-Guided App-Based Virtual Reality Cognitive Behavior Therapy for Acrophobia: A Randomized Controlled Trial
Source: J Clin Med. 2020 May 26;9(6):1614. doi: 10.3390/jcm9061614 (PMC7357041; doi:10.3390/jcm9061614)
Supplement: Supplementary file 1 [file jcm-09-01614-s001.pdf]

Supplementary file 2

Variation in practicing duration for users who experienced at least one VR session

Practice Duration in minutes[1]

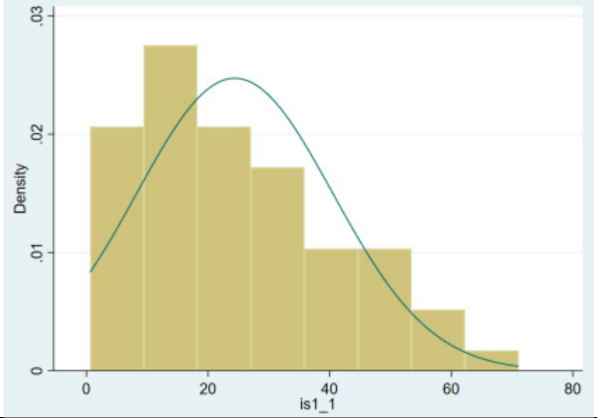

[1]*The plot shows that the distribution is skewed to the right, but smooth and continuous . A formal test for detecting outliers relies on the normality assumption. Notwithstanding this caveat, Grubbs' test confirms the apparent absence of outliers*
